# Supplementary figures and images for: Comparative virulence analysis of seven diverse strains of Orientia tsutsugamushi reveals a multifaceted and complex interplay of virulence factors responsible for disease
Source: PLoS Pathog. 2025 Jun 30;21(6):e1012833. doi: 10.1371/journal.ppat.1012833 (PMC12237263; doi:10.1371/journal.ppat.1012833)

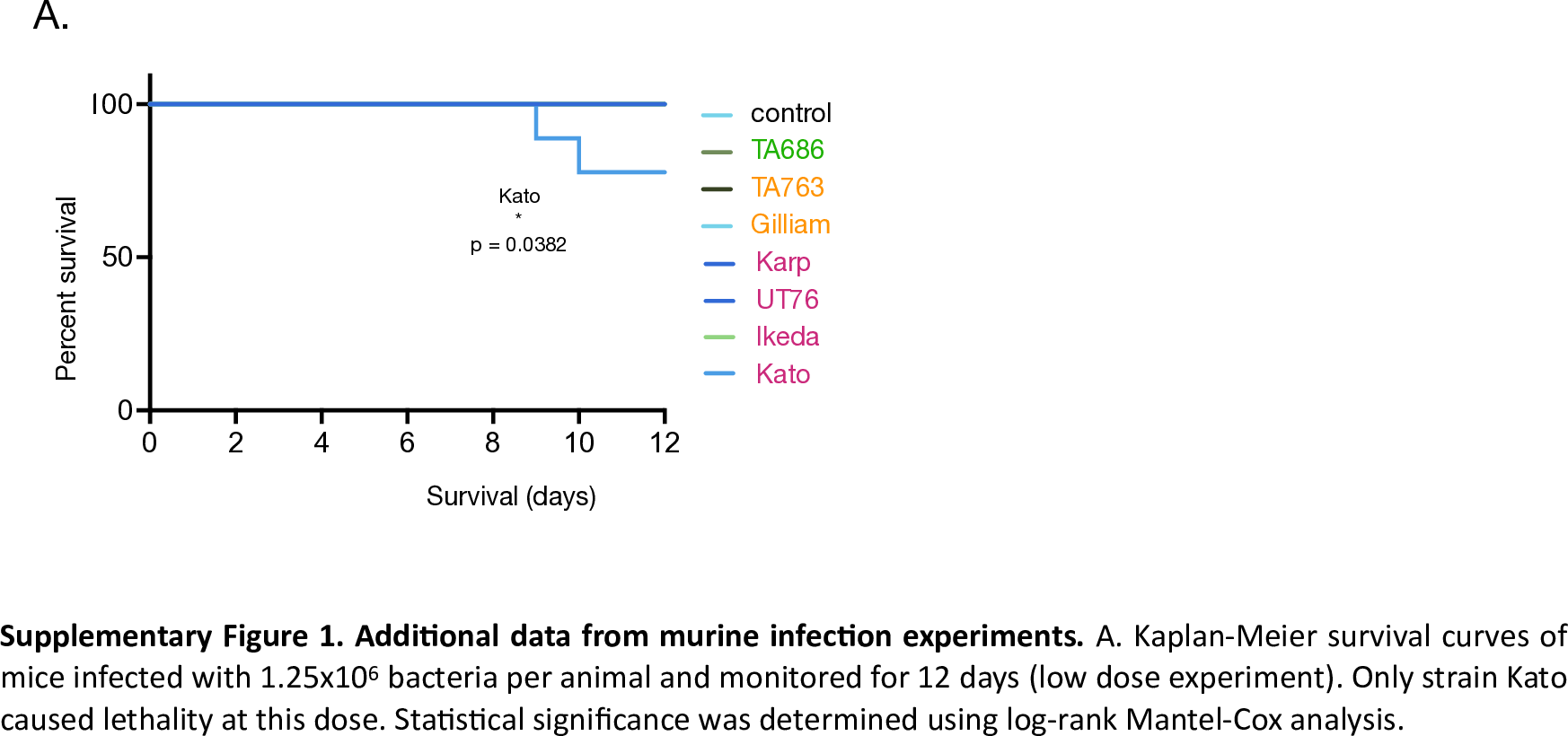

Supplement: S1 Fig — A. Kaplan-Meier survival curves of mice infected with 1.25x10^6^ bacteria per animal and monitored for 12 days (low dose experiment). Only strain Kato caused lethality at this dose. Statistical significance was determined using log-rank Mantel-Cox analysis. (TIF) [file ppat.1012833.s001.tif]

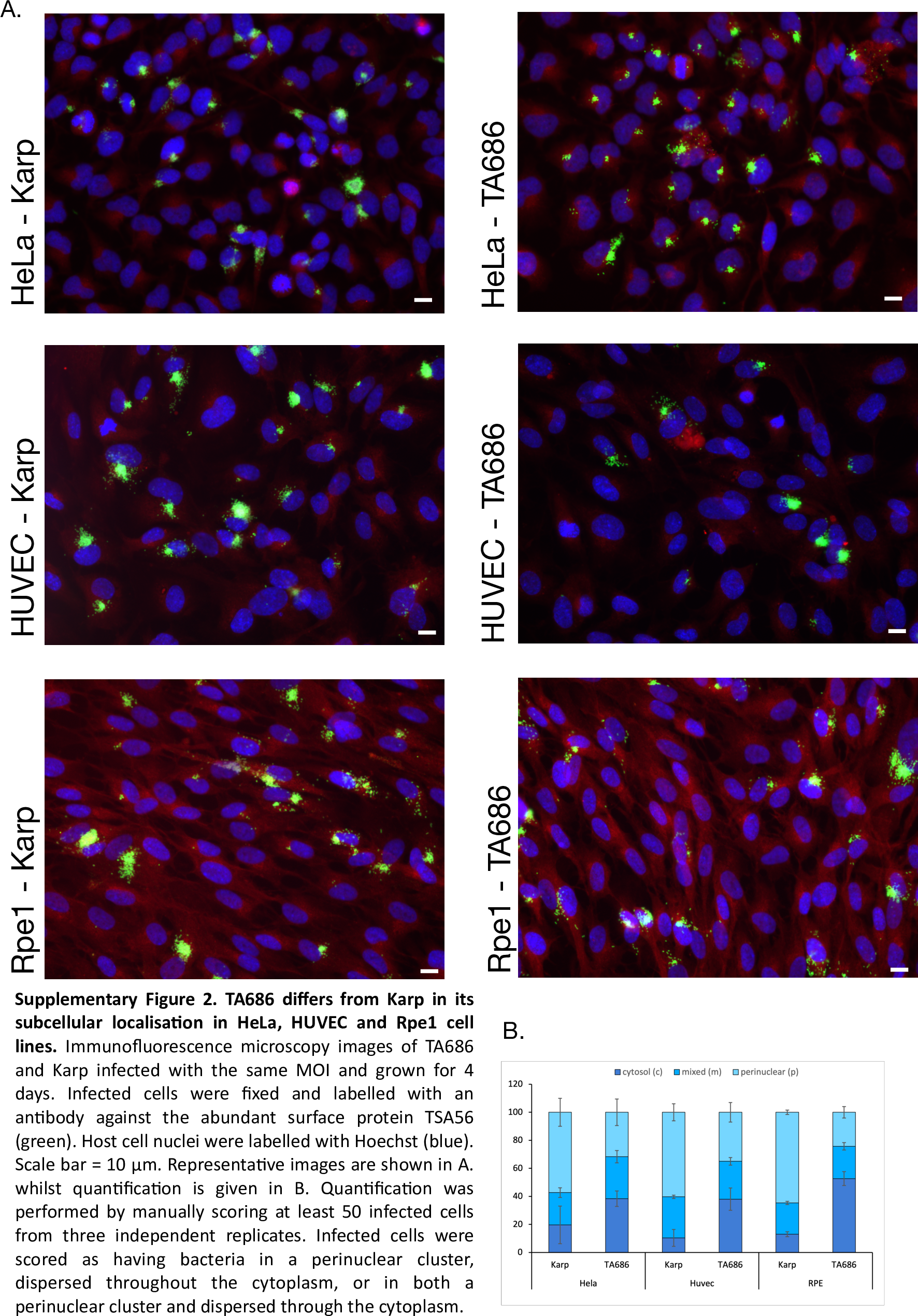

Supplement: S2 Fig — Immunofluorescence microscopy images of TA686 and Karp infected with the same MOI and grown for 4 days. Infected cells were fixed and labelled with an antibody against the abundant surface protein TSA56 (green). Host cell nuclei were labelled with Hoechst (blue). Representative images are shown in A. whilst quantification is given in B. Quantification was performed by manually scoring at least 50 infected cells from three independent replicates. Infected cells were scored as having bacteria in a perinuclear cluster, dispersed throughout the cytoplasm, or in both a perinuclear cluster and dispersed through the cytoplasm. (TIF) [file ppat.1012833.s002.tif]
