# Supplementary material for: Comparative virulence analysis of seven diverse strains of Orientia tsutsugamushi reveals a multifaceted and complex interplay of virulence factors responsible for disease
Source: PLoS Pathog. 2025 Jun 30;21(6):e1012833. doi: 10.1371/journal.ppat.1012833 (PMC12237263; doi:10.1371/journal.ppat.1012833)
Supplement: S1 Table — (DOCX) [file ppat.1012833.s003.docx]

Supplementary Table 1. 16s RNA sequence identity between strains used in this study

|  | Gilliam | Ikeda | Karp | Kato | TA686 | TA763 | UT76 | UT176 |
| --- | --- | --- | --- | --- | --- | --- | --- | --- |
| Gilliam |  | 99.40% | 99.67% | 99.40% | 98.93% | 99.53% | 99.67% | 99.67% |
| Ikeda | 99.40% |  | 99.73% | 100% | 99.27% | 99.60% | 99.73% | 99.60% |
| Karp | 99.67% | 99.73% |  | 99.73% | 99.27% | 99.87% | 100% | 99.87% |
| Kato | 99.40% | 100% | 99.73% |  | 99.27% | 99.60% | 99.73% | 99.60% |
| TA686 | 98.93% | 99.27% | 99.27% | 99.27% |  | 99.13% | 99.27% | 99.27% |
| TA763 | 99.53% | 99.60% | 99.87% | 99.60% | 99.13% |  | 99.87% | 99.73% |
| UT76 | 99.67% | 99.73% | 100% | 99.73% | 99.27% | 99.87% |  | 99.87% |
| UT176 | 99.67% | 99.60% | 99.87% | 99.60% | 99.27% | 99.73% | 99.87% |  |
