# Supplementary material for: Comparative virulence analysis of seven diverse strains of Orientia tsutsugamushi reveals a multifaceted and complex interplay of virulence factors responsible for disease
Source: PLoS Pathog. 2025 Jun 30;21(6):e1012833. doi: 10.1371/journal.ppat.1012833 (PMC12237263; doi:10.1371/journal.ppat.1012833)
Supplement: S2 Table — (DOCX) [file ppat.1012833.s004.docx]

Supplementary Table 2. *Tsa56* sequences from NCBI, as determined by megaBLAST on 1 July 2024.

|  | 100% | 99% | 98% | 95% | 92% | 90% |
| --- | --- | --- | --- | --- | --- | --- |
| Karp | 37 | 137 | 150 | 394 | 682 | 810 |
| TA686 | 0 | 0 | 0 | 1 | 31 | 79 |
| TA763 | 0 | 13 | 23 | 29 | 88 | 245 |
| Gilliam | 11 | 20 | 23 | 374 | 483 | 587 |
| Kato | 16 | 58 | 71 | 99 | 210 | 316 |
| UT76 | 105 | 254 | 374 | 408 | 525 | 709 |
| UT176 | 1 | 41 | 54 | 122 | 286 | 371 |
| Ikeda | 12 | 19 | 20 | 207 | 608 | 643 |
| Boryong | 89 | 229 | 238 | 244 | 354 | 950 |
