# Supplementary material for: Comparative virulence analysis of seven diverse strains of Orientia tsutsugamushi reveals a multifaceted and complex interplay of virulence factors responsible for disease
Source: PLoS Pathog. 2025 Jun 30;21(6):e1012833. doi: 10.1371/journal.ppat.1012833 (PMC12237263; doi:10.1371/journal.ppat.1012833)
Supplement: S3 Table — (DOCX) [file ppat.1012833.s005.docx]

Supplementary Table 3. Nucleotide sequences of oligonucleotide primers for mouse genes

| Cytokine | Forward primers | Reverse primers |
| --- | --- | --- |
| IL-1β | 5’-TCGCTCAGGGTCACAAGAAA-3’ | 5’-ATCAGAGGCAAGGAGGAAACAC-3’ |
| IL-6 | 5’-TAGTCCTTCCTACCCCAATTTCC-3’ | 5’-TTGGTCCTTAGCCACTCCTTC-3’ |
| IL-33 | 5’-TCCAACTCCAAGATTTCCCCG-3’ | 5’-CATGCAGTAGACATGGCAGAA-3’ |
| TNF-α | 5’-ATAGCTCCCAGAAAAGCAAGC-3’ | 5’-CACCCCGAAGTTCAGTAGACA-3’ |
| CCL2/MCP-1 | 5’-GTGCTGACCCCAAGAAGGAA-3’ | 5’-GTGCTGAAGACCTTAGGGCA-3’ |
| CCL4/MIP-1 | 5’-TTCCTGCTGTTTCTCTTACACCT-3’ | 5’-CTGTCTGCCTCTTTTGGTCAG-3’ |
| CCL5/RANTES | 5’-GCTGCTTTGCCTACCTCTCC-3’ | 5’-TCGAGTGACAAACACGACTGC-3’ |
| CXCL9 | 5’-GGAGTTCGAGGAACCCTAGTG-3’ | 5’-GGGATTTGTAGTGGATCGTGC-3’ |
| CXCL10 | 5’-CCAAGTGCTGCCGTCATTTTC-3’ | 5’-GGCTCGCAGGGATGATTTCAA-3’ |
| GAPDH | 5’-TGGAAAGCTGTGGCGTGAT-3’ | 5’-TGCTTCACCACCTTCTTGAT-3’ |
