# Supplementary material for: Comparative virulence analysis of seven diverse strains of Orientia tsutsugamushi reveals a multifaceted and complex interplay of virulence factors responsible for disease
Source: PLoS Pathog. 2025 Jun 30;21(6):e1012833. doi: 10.1371/journal.ppat.1012833 (PMC12237263; doi:10.1371/journal.ppat.1012833)
Supplement: S4 Table — (DOCX) [file ppat.1012833.s006.docx]

Supplementary Table 4. Nucleotide sequences of oligonucleotide primers for human genes

| Cytokine | Forward primers | Reverse primers |
| --- | --- | --- |
| IL-1β | 5’-GCTGAGGAAGATGCTGGTTC-3’ | 5’-TCCATATCCTGTCCCTGGAG-3’ |
| IL-6 | 5’-TACCCCCAGGAGAAGATTCC-3’ | 5’-TTTTCTGCCAGTGCCTCTTT-3’ |
| IL-33 | 5’-CAAAGAAGTTTGCCCCATGT-3’ | 5’-AAGGCAAAGCACTCCACAGT-3’ |
| TNF-α | 5’-CCCGACTATCTCGACTTTGC-3’ | 5’-AGGTTGAGGGTGTCTGAAGGA-3’ |
| CCL2/MCP-1 | 5’-AGGTGACTGGGGCATTGAT-3’ | 5’-GCCTCCAGCATGAAAGTCTC-3’ |
| CCL4/MIP-1 | 5’-GCTTCCTCGCAACTTTGTGG-3’ | 5’-TCACTGGGATCAGCACAGAC-3’ |
| CCL5/RANTES | 5’-CTGCTGCTTTGCCTACATTGC-3’ | 5’-GTTCAGGTTCAAGGACTCTCCATC-3’ |
| CXCL9 | 5’-CCAAGGGACTATCCACCTACAATC-3’ | 5’-GGTTTAGACATGTTTGAACTCCATTC-3’ |
| CXCL10 | 5’-AAGCAGTTAGCAAGGAAAGGTC-3’ | 5’-TTGAAGCAGGGTCAGAACATC-3’ |
| GAPDH | 5’-ATGACAACTTTGGTATCGTGGAAGG-3’ | 5’-GAAATGAGCTTGACAAAGTGGTCGT-3’ |
